# Prioritising target non-pharmacological interventions for research in Parkinson’s disease: achieving consensus from key stakeholders

**DOI:** 10.1186/s40900-020-00212-7

**Published:** 2020-06-24

**Authors:** Angeliki Bogosian, Lorna Rixon, Catherine S. Hurt

**Affiliations:** Division of Health Services Research and Management, City, University of London, London, UK

**Keywords:** Parkinson’s disease, Research prioritisation, Public and patient involvement, Research engagement, Intervention development, Self-management

## Abstract

**Background:**

In 2014 Parkinson’s UK conducted a research prioritisation exercise with stakeholders highlighting important clinical research questions. The exercise highlighted the need for effective interventions to be developed and tested to tackle a range of non-motor symptoms including: sleep quality, stress and anxiety, mild cognitive impairment, dementia and urinary problems. The present work set out to build on this exercise by prioritising types of non-pharmacological interventions to be tested to treat the identified non-motor symptoms.

**Methods:**

A Patient and Public Involvement exercise was used to reach consensus on intervention priorities for the treatment of non-motor symptoms. A Delphi structure was used to support the feedback collected. A first-round prioritisation survey was conducted followed by a panel discussion. Nineteen panellists completed the first-round survey (9 people with Parkinson’s and 10 professionals working in Parkinson’s) and 16 participated in the panel discussion (8 people with Parkinson’s and 8 professionals working in Parkinson’s). A second-round prioritization survey was conducted after the panel discussion with 13 people with Parkinson’s.

**Results:**

Physical activity, third wave cognitive therapies and cognitive training were rated as priority interventions for the treatment of a range of non-motor symptoms. There was broad agreement on intervention priorities between health care professionals and people with Parkinson’s. A consensus was reached that research should focus on therapies which could be used to treat several different non-motor symptoms. In the context of increasing digitisation, the need for human interaction as an intervention component was highlighted.

**Conclusion:**

Bringing together Parkinson’s professionals and people with Parkinson’s resulted in a final treatment priority list which should be both feasible to carry out in routine clinical practice and acceptable to both professionals and people with Parkinson’s. The workshop further specified research priorities in Parkinson’s disease based on the current evidence base, stakeholder preferences, and feasibility. Research should focus on developing and testing non-pharmacological treatments which could be effective across a range of symptoms but specifically focusing on tailored physical activity interventions, cognitive therapies and cognitive training.

## Plain English summary

In 2014 Parkinson’s UK asked people with Parkinson’s, their carers and healthcare professionals working in Parkinson’s, collectively known as stakeholders, to identify aspects of Parkinson’s that urgently needed to be researched to identify new treatments or management strategies. A range of non-motor symptoms of Parkinson’s were ranked as important including: sleep quality, stress and anxiety, mild cognitive impairment, dementia and urinary problems. The purpose of this exercise was to build on the work of Parkinson’s UK by asking a group of stakeholders to identify and prioritise non-drug treatments which should be researched as potential treatments for these non-motor symptoms. This Patient and Public Involvement exercise used a Delphi structure to help reach agreement on which treatments should be prioritised. This consisted of a survey, followed by panel discussion and a post panel survey. Nine people with Parkinson’s and 10 healthcare professionals completed the first round survey, 8 people with Parkinson’s and 8 healthcare professionals participated in the panel discussion and 13 people with Parkinson’s completed the second round survey. There was good agreement on research priorities between people with Parkinson’s and Healthcare professionals. Physical exercise, talking therapies and cognitive training were identified as treatments which had shown some promising improvements in relevant symptoms, were acceptable to people with Parkinson’s and were practical to carry out and therefore should be the focus of research. There was agreement that treatments which had the potential to improve multiple symptoms such as talking therapies should be prioritised. The exercise provides a comprehensive list of practical and acceptable non-drug treatments for non-motor symptoms of Parkinson’s which can be used to push forward research to improve the lives of people with Parkinson’s and their families.

## Background

In 2014 Parkinson’s UK conducted a priority setting exercise to identify research questions that key stakeholders, people with Parkinson’s and clinicians, wanted to prioritise [1]. The exercise highlighted the need to identify and test effective treatments for a range of non-motor symptoms of Parkinson’s disease (PD) including stress and anxiety, dementia, mild thinking and memory problems, sleep and urinary problems. The present work further developed this by asking stakeholders to prioritise potential treatment types for the non-motor symptoms highlighted in the 2014 exercise.

PD is considered to be a movement disorder defined by the presence of motor symptoms, such as bradykinesia, tremor and rigidity. It is now, however, widely accepted that PD is characterised not only by its motor aspects, but also by numerous non-motor symptoms that encompass sensory abnormalities, behavioural changes, sleep disturbances, autonomic dysfunction, and fatigue. In two recent studies, at least one non-motor symptom was reported by almost 100% of patients [2]. The non-motor symptoms of PD can be as disabling for an individual as their motor symptoms, if not more so [3]. Indeed, non-motor symptoms dominate the clinical picture of PD and contribute to severe disability, impaired quality of life, and shortened life expectancy [4, 5].

There is currently limited evidence for effective treatments for non-motor symptoms [6, 7], either pharmacological or non-pharmacological. Consequently, even when non-motor symptoms are recognised in a clinical consultation, treatment rates remain low as evidenced in recent reports [8, 9].

The failure to treat non-motor symptoms due to the lack of effective pharmacological treatments is especially true in the case of fatigue, anxiety and depression in PD. People with PD benefit less from antidepressant treatment, than do people without PD [10]. Also, there is a high risk of adverse side effects and adverse interactions between antidepressants and antiparkinsonian medications [11]. Benzodiazepines, used commonly for anxiety disorder treatment, are not recommended for people living with PD due to adverse effects including cognitive and psychomotor impairment [12] and increased risk of falls [13]. Atomoxetine, was not found to be efficacious for anxiety in PD [14]. Currently insufficient evidence exists to support the treatment of fatigue in PD with any drug or non-pharmacological treatment, highlighting the need for further research [15]. Furthermore, there is often a reluctance by many PD patients to take additional medication or change finely balanced medication regimes for motor symptoms in order to treat non-motor symptoms [16].

For non-motor symptoms where pharmacological treatments lack effectiveness, there is a growing evidence base showing that non-pharmacological treatments might be able to help. Cognitive Behavioural Therapy (CBT), including distance delivered CBT, has moderate effects on improving anxiety and depression, insomnia and impulse-control disorders in PD [17, 18]. Emerging evidence has suggested that mindfulness-based interventions can help reduce symptoms of depression [19–21], and symptoms of anxiety [19, 21]. Consequently the present exercise sought to prioritize non-pharmacological treatments for a range of non-motor symptoms idenitfied as priorities in the 2014 exercise, namely: stress and anxiety, dementia, mild thinking and memory problems, sleep and urinary problems.

## Methods

### Structure

**Fig. 1:**
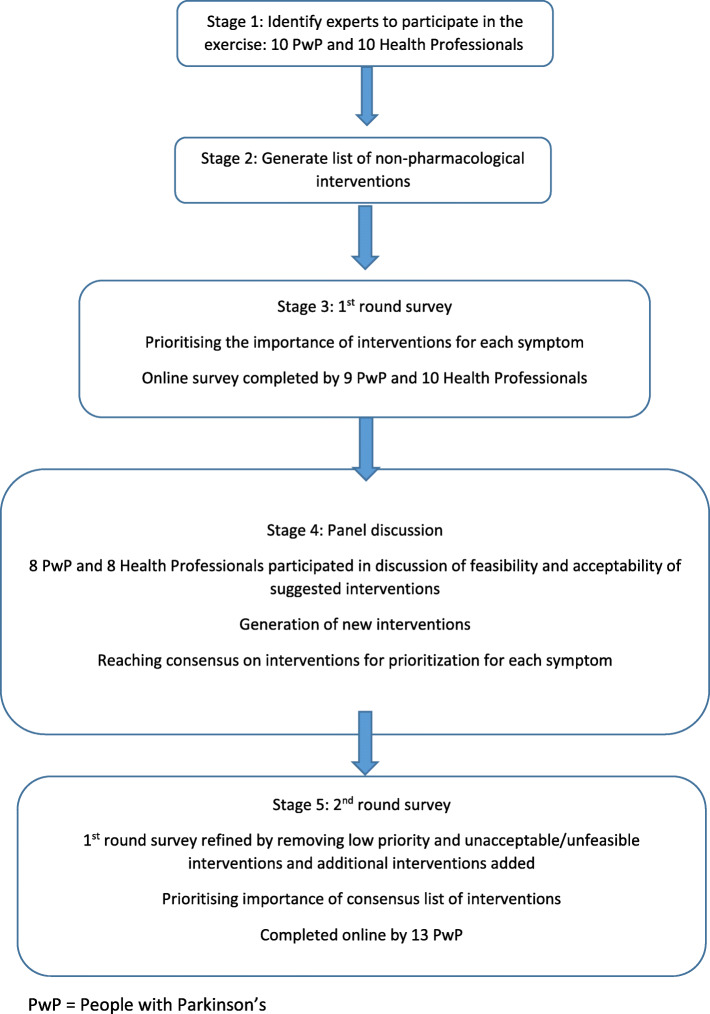
Exercise Flow chart

From the outset of this Patient and Public Involvement (PPI) exercise we decided to adopt elements of the Delphi technique to guide the development of consensus. The purpose of the meeting was to bring together relevant stakeholders to identify and prioritise psychological and behavioural interventions which may improve non-motor symptoms. Using guidance from the Delphi technique helped us to collect stakeholders’ feedback in a more systematic way. The Delphi technique is an iterative survey exercise with controlled feedback to a group of panellists [22]. The ‘panellists’ are purposively invited for their particular expertise on a topic and the surveys are often conducted across a series of two or more sequential ‘rounds’. In the current prioritisation exercise, two rounds of surveys were used; one before and one following the panel discussion. The structure of the process is outlined in Fig. 1.

We brought together a range of key stakeholders: people with Parkinson’s, psychological and behavioural researchers specialising in Parkinson’s, and healthcare professionals working in Parkinson’s. Bringing together all interested parties in a single day meeting allowed dialogue between individuals and the sharing of perspectives to ensure that decisions regarding the final research priorities were collaborative.

### Stage 1: identifying experts for the exercise ‘the panel’

Turoff [23] recommends panels between 10 and 50. Ten people with Parkinson’s and ten health professionals (geriatrician, psychologists, PD nurses, physiotherapist, occupational therapist, speech therapist) initially agreed to take part in the exercise. The health professionals, whose expertise was based on qualifications and proven track records in the field, were identified through peer consultation and invited via email by the authors. People with Parkinson’s and carers were invited by Parkinson’s UK through an email to their Research Network mailing list.

### Stage 2: generate list of non-pharmacological interventions

The initial list of non-pharmacological interventions for the first round survey was developed from literature reviews in PD and similar conditions conducted by two authors (AB and LR) who specialise in behavioural interventions in PD. Due to resource constraints the panellists were not consulted in this initial idea generation phase for salient non-pharmacological interventions to include in the survey.

### Stage 3: survey round 1

**Fig. 2:**
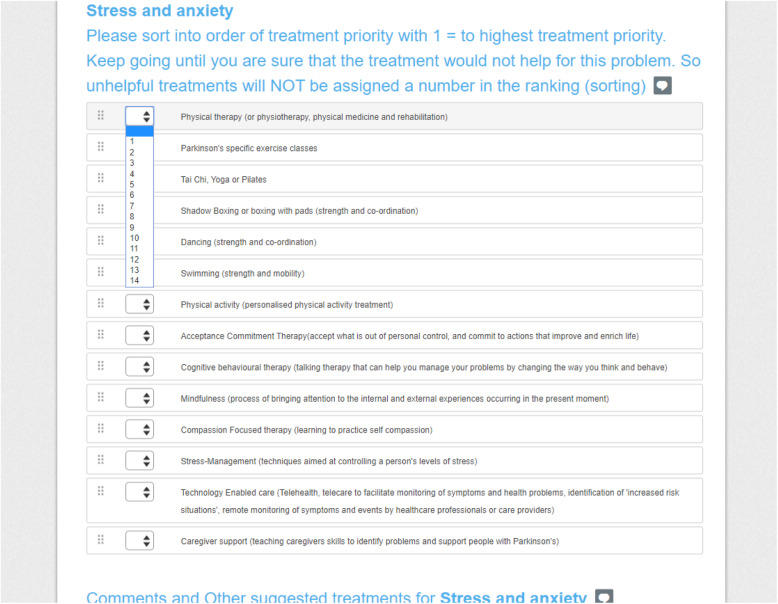
Treatment Priority Ranking in the Round One Survey

The survey rounds were completed using the online tool Survey Monkey [24]. The survey asked panellists to rank the importance of each suggested non-pharmacological intervention for each of the non-motor symptoms identified as research priorities in the Parkinson’s UK prioritization exercise: stress and anxiety, dementia, mild thinking and memory problems, sleep and urinary problems) [1]. A short explanation of each intervention was provided for clarity. Panellists were asked to rank the interventions into order of treatment priority with 1 = highest treatment priority using a drop-down menu. Respondents were then instructed to keep assigning numbers to each treatment until they were sure that the treatment would not help for the symptom. Unhelpful treatments were not assigned a number in the ranking. A screen shot of the treatment ranking exercise is shown in Fig. 2.

### Stage 4: the panel discussion in-between survey rounds

The panel discussion was facilitated by the first and second author. All members of the panel were made aware of the background of the two facilitators, i.e. health psychology researchers in the area of non-pharmacological treatments in PD and were also made aware of their interest, i.e. identify research priorities for future research grant applications. Respondents were aware of the topic of the discussion and had already taken part in the survey that the discussion was based on. No other preparation was required prior to the meeting. The panel discussion included eight people with Parkinson’s, and eight health professionals. The discussion started with a short presentation on the most prevalent non-motor symptoms, followed by our suggestions for non-pharmacological interventions, followed by the ranking results of the first-round survey. The non-motor symptom priorities focused on during the workshop were: stress and anxiety, dementia, mild cognitive problems, quality of sleep, urinary problems. For each of these categories, panellists were asked to discuss:

- What behavioural and psychological management interventions are available?
- What is the research evidence and your personal experience with management of these non-motor symptoms?
- Which behavioural and psychological management interventions do you consider as the highest priorities?

The group discussed preferences in terms of types of psychological and behavioural interventions for non-motor symptoms in Parkinson’s, as well as the mode of delivery, that best suits people with Parkinson’s and how likely these interventions were to be translated into clinical practice. Current clinical practices in Parkinson’s were also discussed and how potential interventions on specific non-motor symptoms could be added to current common practice.

The group then prioritised interventions for research based on potential intervention efficacy, acceptability, need and translation into clinical practice. After discussing specific treatments, the facilitators asked the group which non-pharmacological intervention they consider the most important and promising. One of the panellists kept notes on a white board as people offered suggestions and thoughts. This discussion largely focused on one specific intervention and the facilitators summarised and confirmed with the group that this intervention should be prioritised.

### Stage 5: survey round 2

Following the panel discussion, the first-round survey was refined by adding interventions not previously included and narrowing down the available intervention options for each non-motor symptom. We removed interventions with very low rankings at the first survey or interventions that were not considered appropriate based on the panel discussion. For example, we added ‘peer group support’ under ‘anxiety’ and removed ‘acceptance and commitment therapy’ under ‘sleep’. A question about mode of delivery preferences was also added.

For the second-round survey we asked the panel to re-rate the interventions suggested for each non-motor symptom and emailed the survey to Parkinson’s UK Research Network members. Thirteen people with Parkinson’s responded to the survey, two of whom had attended the workshop.

### Ethics

The goal of the exercise was to gather information to direct future research using Public and Patient Involvement. According to NIHR INVOLVE guidelines ethical approval is not needed when the public acts as specialist advisors, providing expertise based on their experience of a health condition in planning or advising on research. Prior published research priority setting exercises have also suggested that ethical approval is not required [1]. It was assumed that the ability to complete the online surveys suggested that the respondents had capacity to consent in the exercise. No incentives were offered to respondents but all travel expenses were reimbursed.

## Results

### Intervention ranking

The results of the first-round survey showed that physical activity, stress-management and cognitive training were high priorities for a variety of non-motor symptoms. More details on the top three behavioural interventions for each non-motor symptom are presented in Table 1. Physical activity, cognitive training and third wave therapies including cognitive behaviour therapy and mindfulness were all ranked as high priorities in the second-round survey.

**Table 1:** Top three non-pharmacological interventions identified for each non-motor symptom in the first and second round surveys

Non-motor symptom	Interventions (1st round survey)	Panel discussion (consensus following discussion of efficacy, acceptability and need)	Interventions (2nd round survey)
Stress and anxiety	1. Cognitive behavioural therapy (talking therapy that can help you manage your problems by changing the way you think and behave) 2. Stress management (techniques aimed at controlling a person’s levels of stress) 3. Mindfulness	1. Acceptance and commitment therapy (acceptance and committing to valued actions) 2. Mindfulness 3. Cognitive behavioural therapy (talking therapy that can help you manage your problems by changing the way you think and behave)	1. Physical activity (personalised physical activity) 2. Cognitive behavioural therapy (talking therapy that can help you manage your problems by changing the way you think and behave) 3. Mindfulness
Dementia	1. Cognitive skills training (compensatory cognitive skills from neurorehabilitation) 2. Caregiver support (teaching caregivers skills to identify problems and support people with Parkinson’s) 3. Lifestyle management strategies	1. Screening programme to detect /monitor cognitive changes. 2. Cognitive skills training (compensatory cognitive skills from neurorehabilitation) 3. Caregiver support (teaching caregivers skills to identify problems and support people with Parkinson’s)	1. Lifestyle management strategies 2. Caregiver support (teaching caregivers skills to identify problems and support people with Parkinson’s) 3. Cognitive skills training (compensatory cognitive skills from neurorehabilitation)
Mild thinking and memory problems	1. Cognitive skills training (compensatory cognitive skills from neurorehabilitation) 2. Stress management (techniques aimed at controlling a person’s levels of stress) 3. Physical activity (personalised physical activity treatment)	1. Screening programme to detect /monitor cognitive changes. 2. Cognitive skills training (compensatory cognitive skills from neurorehabilitation) Caregiver support (teaching caregivers skills to identify problems and support people with Parkinson’s)	1. Physical activity (personalised physical activity treatment) 2. Cognitive skills training (compensatory cognitive skills from neurorehabilitation) 3. Stress management (techniques aimed at controlling a person’s levels of stress)
Quality of sleep	1. Sleep hygiene 2. Physical activity (personalised physical activity treatment) 3. Mindfulness	1. Sleep hygiene 2. Technology Enabled care (to monitor sleep, but also falls and nocturia at night)	1. Sleep hygiene 2. Physical activity (personalized physical activity treatment) 3. Mindfulness
Urinary problems	1. Self-management for urinary problems (fluid management, caffeine and alcohol management, bladder retraining) 2. Lifestyle management strategies 3. Technology Enabled care	1. Self-management for urinary problems (fluid management, caffeine and alcohol management, bladder retraining)	1. Self-management for urinary problems (fluid management, caffeine and alcohol management, bladder retraining) 2. Lifestyle management strategies 3. Technology Enabled care

§ = includes physiotherapy and rehabilitation §§ = exercise classes and program

During the panel discussion additional behavioural interventions were discussed, such as peer support groups to manage stress and anxiety; on-going assessments and care for dementia, pelvic floor exercises as part of self-management for urinary problems and massage and the use of a light box to help manage sleep. These non-pharmacological interventions were added in the post-panel survey, but they were not identified as a priority (Table 1). Table 2 summarizes the number of interventions that were added and removed at each stage of the process.

**Table 2:** Number of non-pharmacological interventions in advance of, during and after the panel discussion

Key non-motor symptoms	Total number of interventions generated at pre-discussion survey	Number of interventions remaining following panel discussion	Additional interventions generated during discussion	Final number of interventions for ranking
*Stress and anxiety*	14	7	1	8
*Dementia*	17	5	1	6
*Mild thinking and memory problems*	17	7	0	7
*Quality of sleep*	17	5	2	7
*Urinary problems*	4	0	0	4

As shown in Table 1, the three highest ranked interventions for each non-motor symptom did not change significantly between the first and second survey rounds. Ten HCPs and 9 people with Parkinson’s responded to the first-round survey and 13 people with Parkinson’s responded to the second-round survey. Table 3 shows the first-round survey responses divided by respondent type (professional vs person with Parkinson’s). There was broad agreement on intervention priorities across respondent group.

**Table 3:** First round survey priorities by respondent type

Key non-motor symptoms	Professionals top three interventions	People with Parkinson’s top three interventions
*Stress and anxiety*	1. Cognitive behavior therapy	1. Cognitive behavior therapy
	2. Mindfulness	2. Stress management
	3. Stress management	3. Mindfulness
*Dementia*	1. Cognitive skills training	1. Cognitive skills training
	2. Lifestyle management strategies	2. Carer support
	3. Carer support	3. Compassion focused therapy
*Mild thinking and memory problems*	1. Cognitive skills training	1. Cognitive skills training
	2. Acceptance and commitment therapy	2. Cognitive behavior therapy
	3. Stress management	3. Physical activity
*Quality of sleep*	1. Sleep hygiene	1. Physical activity
	2. Cognitive behavior therapy	2. Sleep hygiene
	3. Self-management	3. Mindfulness
*Urinary problems*	1. Self-management	1. Self-management
	2. Lifestyle management	2. Lifestyle management
	3. Carer support	3. Carer support

### Mode of delivery

In the second-round survey a question was added exploring preferences for mode of delivery of non-pharmacological interventions. Of the 13 PD respondents, eight preferred individual face-to-face delivery of interventions, four preferred online delivery with some peer or professional contact, and one wanted group support or group therapy.

### Outcomes from the panel discussion

There was consensus that physical exercise is beneficial in PD but there is limited knowledge on PD specific exercises. There was also a consensus that ideally, we need an intervention that will cover more than one symptom. For example, talking therapies could be applied to more than one non-motor symptom at a time, such as anxiety, depression, and sleep problems, and augment other treatment approaches, such as facilitating adherence to exercise, pacing activities of daily living and self-management.

People with Parkinson’s emphasised the need for personalised treatments. They were aware that one size did not fit all and that the same symptoms can impact people differently, so they needed to be cautious when suggesting one treatment for one symptom in all cases. In order to get the maximum potential benefit from treatments delegates agreed that treatments need to be tailored to the individual.

With the increasing use of digital technologies to deliver interventions the panel reached a consensus that face to face contact in intervention delivery remained of central importance as a method of combating social isolation.

## Discussion

This exercise extended the priority setting work conducted by Parkinson’s UK [4] with a focus on prioritising non-pharmacological treatments to tackle the non-motor symptoms highlighted by the Parkinson’s UK exercise namely: sleep quality, dementia, mild memory problems, stress and anxiety and urinary problems.

There was good consensus on treatment priorities between Parkinson’s professionals and people with Parkinson’s. Many overlapping interventions were identified for different symptoms for example physical activity, cognitive skills training and mindfulness. While both people with PD and healthcare professionals generally ranked physical activity as a priority it was evident that there was a lack of clarity around which physical exercises were recommended for people with Parkinson’s. Research to date has shown physical exercise to have beneficial effects on a range of non-motor symptoms [25]. Future research should focus on providing evidence-based guidance for physical activity in PD that can be easily implemented by clinicians and patients.

Similarly, there is accumulating evidence for the efficacy of cognitive skills training in PD [26] but there is large methodological variability between studies and a limited understanding of the long-term efficacy of this approach. Future research should seek to conduct larger, controlled studies which aim to determine which patient groups may benefit most from cognitive skills training [26] enabling targeted provision for those who will benefit most.

The efficacy of third wave therapies such as mindfulness, cognitive behaviour therapy and stress management is increasingly being tested for a range of non-motor symptoms in PD [19–21, 27–29] with some positive preliminary results. Large, controlled trials with longer follow up periods are needed.

A challenge of providing these interventions is often one of resource, particularly when a trained therapist is required to implement an intervention. Despite the recent proliferation of online interventions in Parkinson’s [27, 28] which have clear practical benefits, there was a consensus that an element of face-to-face contact was required in intervention delivery. It is essential to carefully balance the preferences of people with Parkinson’s with the practicalities of delivering cost-effective interventions to large groups. Consequently, finding innovative ways to implement the intervention whilst still maintaining human contact, rather than taking a purely digital approach, is paramount. Recent work exploring the use of skype conferencing to deliver mindfulness interventions [27, 30] or the use of lay facilitators to deliver interventions across conditions may be important avenues for further research [31–33].

It was evident from the panel discussion that rather than treatments tailored to symptoms, people with Parkinson’s and professionals working in the area of Parkinson’s wanted global interventions which might have positive effects across a range of symptoms. Future research should endeavour to explore the use of therapies such as CBT and mindfulness to support self-management of other non-motor symptoms e.g. urinary symptoms and cognitive symptoms.

Bringing together Parkinson’s professionals and people with Parkinson’s allowed both parties views’ to be heard, combining feasibility of delivering an intervention with patient and carer preferences. Asking stakeholders to produce a consensus list of priority interventions helps ensure that the research agenda moves forward and research into identified interventions is undertaken as stakeholders are engaged with the research process. The final treatment priority list should be both feasible to carry out in routine clinical practice and acceptable to both professionals and people with Parkinson’s increasing the likelihood of implementation of effective interventions in the NHS. Furthermore, the bringing together of clinicians, researchers and people with PD provides strategic alliances facilitating future research programmes.

This priority setting exercise was not without limitations. The largest of these being the difference in respondents completing the survey pre and post the panel discussion. The first-round survey was 50% people with Parkinson’s and 50% professionals, the majority of whom then attended the panel discussion. However, the second-round survey was solely completed by people with Parkinson’s, only a small proportion of whom attended the discussion. Possible reasons for the lack of engagement in the second-round survey could include the realities of a busy schedule or panellists may have felt that they had ‘already had their voice heard’. This latter point may have been more salient in the present exercise as relatively few changes were made to the intervention list as a consequence of the panel discussion. Therefore panellists may have felt the process had an element of repetition.

It is possible that the interventions prioritized in the second-round survey only reflect the views of people with Parkinson’s as no professionals completed this round. However, the concordance in priority setting seen between professionals and people with Parkinson’s in the first-round survey suggests that the second-round survey results may have been relatively similar had it also been completed by Parkinson’s professionals. Conversely the inclusion of a largely new group of respondents in the second-round survey provides support for the generalisability of the findings in the first round of the survey. Treatment priorities showed little variation pre and post panel discussion.

## Conclusions

In summary, the present exercise further specifies research priorities in Parkinson’s disease based on the current evidence base, stakeholder preferences, and feasibility. Research should focus on developing and testing non-pharmacological treatments which could be effective across a range of non-motor symptoms but specifically focusing on tailored physical activity interventions, cognitive skills training and psychological therapies including mindfulness, cognitive behavioural therapy and stress management.

## Data Availability

The datasets used and analysed during the current study are available from the corresponding author on reasonable request.

## Abbreviations

CBT: Cognitive behavioural therapy
HCP: Health Care Professional
PD: Parkinson’s disease
PPI: Patient and public involvement
PwP: Person with Parkinson’s
